# A Theory and Evidence-Based Artificial Intelligence-Driven Motivational Digital Assistant to Decrease Vaccine Hesitancy: Intervention Development and Validation

**DOI:** 10.3390/vaccines12070708

**Published:** 2024-06-25

**Authors:** Yan Li, Kit-Ching Lee, Daniel Bressington, Qiuyan Liao, Mengting He, Ka-Kit Law, Angela Y. M. Leung, Alex Molassiotis, Mengqi Li

**Affiliations:** 1 School of Nursing, The Hong Kong Polytechnic University, Hong Kong 999077, China; yan-nursing.li@polyu.edu.hk (Y.L.); kching.lee@polyu.edu.hk (K.-C.L.); mandy-mt.he@polyu.edu.hk (M.H.); ka-kit.law@polyu.edu.hk (K.-K.L.); angela.ym.leung@polyu.edu.hk (A.Y.M.L.); 2 Faculty of Health, Charles Darwin University, Darwin 0815, Australia; daniel.bressington@cdu.edu.au; 3 School of Public Health, The University of Hong Kong, Hong Kong 999077, China; qyliao11@hku.hk; 4 Research Institute for Smart Aging (RISA), The Hong Kong Polytechnic University, Hong Kong 999077, China; 5 College of Arts, Humanities and Education, University of Derby, Derby DE22 1GB, UK; a.molasiotis@derby.ac.uk

**Keywords:** vaccine hesitancy, artificial intelligence, chatbot, motivational interviewing, COVID-19

## Abstract

Vaccine hesitancy is one of the top ten threats to global health. Artificial intelligence-driven chatbots and motivational interviewing skills show promise in addressing vaccine hesitancy. This study aimed to develop and validate an artificial intelligence-driven motivational digital assistant in decreasing COVID-19 vaccine hesitancy among Hong Kong adults. The intervention development and validation were guided by the Medical Research Council’s framework with four major steps: logic model development based on theory and qualitative interviews (n = 15), digital assistant development, expert evaluation (n = 5), and a pilot test (n = 12). The Vaccine Hesitancy Matrix model and qualitative findings guided the development of the intervention logic model and content with five web-based modules. An artificial intelligence-driven chatbot tailored to each module was embedded in the website to motivate vaccination intention using motivational interviewing skills. The content validity index from expert evaluation was 0.85. The pilot test showed significant improvements in vaccine-related health literacy (*p* = 0.021) and vaccine confidence (*p* = 0.027). This digital assistant is effective in improving COVID-19 vaccine literacy and confidence through valid educational content and motivational conversations. The intervention is ready for testing in a randomized controlled trial and has high potential to be a useful toolkit for addressing ambivalence and facilitating informed decision making regarding vaccination.

## 1. Introduction

The World Health Organization (WHO) estimates that vaccination annually prevents 3.5 to 5 million deaths from life-threatening diseases such as diphtheria, tetanus, pertussis, influenza, and measles [1]. Vaccine popularization is widely considered one of the most cost-effective measures against infectious diseases [2]. The World Health Assembly endorsed the Immunization Agenda 2030, a global initiative that aims to save more than 50 million lives from vaccine-preventable diseases in the next decade [3]. However, vaccine hesitancy, defined as a delay in acceptance or refusal of vaccines despite their availability, remains a significant barrier to achieving the full potential of vaccination [4]. Vaccine hesitancy, a widespread phenomenon occurring with a variety of existing vaccines, is not new; however, it particularly manifests with newly developed vaccines [5]. A recent systematic review suggested that the vaccine hesitancy rate was 23.3% for diphtheria and tetanus and 45.3% for measles [6]. An international survey conducted between November 2020 and January 2021 found a high prevalence (33.3% to 85%) of COVID-19 vaccine hesitancy across 13 countries (i.e., Australia, Brazil, Canada, Chile, China, Colombia, France, India, Italy, Spain, Uganda, UK, and the USA) [7].

Vaccine hesitancy is a complex and multifaceted issue, influenced by diverse individual, group, and environmental factors, extending beyond mere informational gaps [5]. Developing effective interventions to address vaccine hesitancy is crucial for global health promotion, yet it remains a significant challenge despite extensive efforts that have been made [8]. The WHO EURO Vaccine Communications Working Group proposed the “3 Cs” model (i.e., confidence, complacency, and convenience) to conceptualize the complex psychosocial factors underlying vaccine hesitancy [4]. The primary barriers to vaccine acceptance identified from systematic reviews include concerns about vaccine safety and efficacy (confidence), perceived low severity and susceptibility of infection (complacency), and perceived low availability (convenience) [9]. Educational interventions delivered via education booklets, phone calls, and PowerPoint presentations have been developed to improve vaccine confidence, reduce complacency, and enhance the perceived convenience of taking vaccines [10,11,12]. However, relying solely on didactic education proves insufficient in effectively inducing behavior change. Motivational interviewing (MI) is an effective and evidence-based psychological counseling technique that utilizes collaborative conversations [13]. It has demonstrated great benefits in addressing ambivalence and enhancing motivation and commitment to change [14]. MI skills have been utilized by healthcare workers and shown effectiveness in reducing vaccine hesitancy by addressing individual concerns and empowering personal agency to vaccinate [15,16]. MI skills can be a promising approach incorporated with education to effectively address vaccine hesitancy [17].

Additional attempts have been made to utilize tele-interventions or digital platforms to offer accessible support in addressing vaccine hesitancy. These interventions are delivered in a variety of modalities, including videoconferencing, phone calls, and web-based interventions [11,12,18,19]. Artificial intelligence (AI)-driven chatbots are emerging tools used in health services [20]. With natural language processing technology, an AI-driven chatbot can provide a promising e-health platform that may benefit a wide range of users simultaneously, conserve healthcare resources and time, and ensure privacy protection [21]. Chatbots have demonstrated effectiveness in significantly increasing individuals’ intentions to get vaccinated against COVID-19 in the French populations through engaging conversations compared to reading educational materials [22]. By incorporating therapeutic dialogues with MI skills into the chatbot, the motivational chatbot can be empathetic to embrace resistance and support self-efficacy during conversations, contributing to a significant increase in motivation to quit smoking [23]. However, the integration of MI skills and AI-driven chatbots applied in addressing vaccine hesitancy has not been explored to date.

Vaccine hesitancy was found to be particularly prevalent in high-income countries or regions [7]. Hong Kong is one of these regions with a high COVID-19 vaccine hesitancy of 45.3% in 2022, as indicated in a population-based survey conducted during the fifth wave of COVID-19 in Hong Kong [24]. Given the complex and contextual nature of vaccine hesitancy, it is recommended to develop accessible, effective, and tailored interventions that align with local context and individual needs [25]. This paper reports on the development and validation of a novel, tailored, AI-driven motivational digital assistant that incorporates web-based education and an AI-driven chatbot equipped with MI skills. This digital assistant was hypothesized to be acceptable and preliminarily effective in decreasing COVID-19 vaccine hesitancy among Hong Kong adults.

## 2. Materials and Methods

### 2.1. Overview

**Figure 1:**
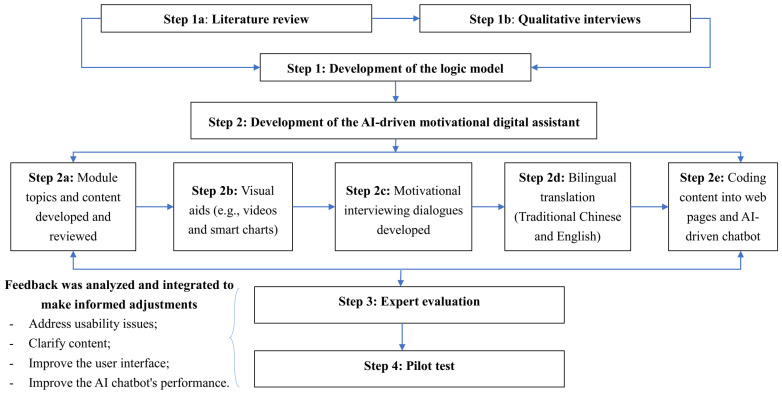
The intervention development and validation processes.

An AI-driven motivational digital assistant “Auricle” in addressing vaccine hesitancy was developed by a multidisciplinary team of researchers in vaccines, psychology, and computer science. We followed the best practices to conduct intervention development and validation, as recommended by the Medical Research Council’s guidance [26]. The intervention development and validation involve systematic processes integrating theory- and empirical evidence-based approaches, which are described in detail as the following four steps (see Figure 1): (1) the development of the logic model; (2) the development of the AI-driven motivational digital assistant; (3) expert evaluation; and (4) a pilot test. This study was approved by the Human Subjects Ethics Review Board of Hong Kong Polytechnic University (HSEARS20210813003). Written informed consent was obtained from all participants.

### 2.2. Step 1: The Development of the Logic Model

Depicting the intervention in a logic model helps to clarify causal hypotheses and mechanisms of the intervention’s impact, as indicated by the Medical Research Council’s guidance [27]. The logic model schematically demonstrates the relationships among (1) intervention inputs; (2) intervention activities; (3) key mediators; and (4) primary and secondary outcomes. In this study, the logic model was developed by adopting two approaches. First, a literature review of relevant theoretical frameworks was conducted to identify the factors associated with vaccine hesitancy. Then, a qualitative study was conducted to explore the specific factors influencing COVID-19 vaccine hesitancy among Hong Kong residents.

The Vaccine Hesitancy Determinants Matrix Model, developed by the WHO Strategic Advisory Group of Experts on Immunization, was utilized as a comprehensive theoretical framework in this study to understand the influencing factors of vaccine hesitancy [4]. This model is built on systematically reviewed studies and the working group’s expertise [28]. It has been widely used in different countries or regions to guide research exploring factors that influence vaccine hesitancy [29,30]. The model lists three categories of factors: contextual influences, arising due to historic, sociocultural, environmental, health system/institutional, economic, or political factors; individual and group influences, arising from personal perception or social/peer environment influences; and vaccine/vaccination-specific issues, directly related to vaccines or vaccination [4].

As guided by the Vaccine Hesitancy Determinants Matrix Model, we developed an interview guide to explore how three categories of factors may influence Hong Kong adults’ attitudes toward COVID-19 vaccination. One-to-one semi-structured interviews were conducted with Hong Kong adults from March to May 2022. Hong Kong residents aged 18 years or older who were hesitant towards COVID-19 vaccines (i.e., not taking COVID-19 vaccines or receiving involuntary COVID-19 vaccines) and able to communicate in Cantonese or English were considered eligible for interview. A purposive sampling method was used to ensure maximum variation by sampling participants from different backgrounds and conditions (i.e., age, gender, ethnicity, education level, and work experience) [31]. The sample size was determined until information saturation was reached as indicated by no new categories being identified in the data [32]. An experiment suggested that data saturation occurred within the first twelve interviews [33]. Content analysis was used to analyze interview data, and this was conducted concurrently with data collection [34].

### 2.3. Step 2: The Development of the AI-Driven Motivational Digital Assistant

The AI-driven motivational digital assistant “Auricle”, incorporating a web-based educational program with a motivational AI-driven chatbot, was developed from October to December 2022. Web-based modules were specifically designed to address COVID-19 vaccine hesitancy among Hong Kong adults, offering informative educational resources. An AI-driven chatbot combined with MI skills was incorporated into web-based modules to provide therapeutic dialogues and instant responses for vaccine-related questions, aiming at improving motivation for taking vaccines.

To develop the AI-driven motivational digital assistant, a multi-step process was followed: (1) Module topics (e.g., myths about COVID-19 vaccines) were identified based on the findings generated in step 1. Module contents (e.g., common myths/rumors regarding vaccine safety and efficacy) for each topic and their adaptations for conversational Q&A were developed and reviewed by members of the research team. To enhance the trustworthiness of module content, two research team members (Kit-Ching Lee and Mengting He) conducted extensive data searches from medical databases (e.g., MEDLINE), the WHO’s COVID-19 special website [35], Hong Kong government websites [36], and other official sources to identify relevant information and receive regular updates. Multiple-choice questions were designed for each module to encourage reflection, interaction, and engagement. (2) Visual aids (e.g., videos and smart charts) were used to visualize the text information to improve readability and engagement. (3) MI dialogues tailored to each module were developed by two research team members (Daniel Bressington and Ka-Kit Law) with a psychology background and motivational interviewing experiences. The four processes of “Engaging, Focusing, Evoking, and Planning” were used to guide the development of MI dialogues [13]. The four principles, including expressing empathy, developing discrepancy, rolling with resistance, and supporting self-efficacy, were incorporated into the dialogues to initiate motivation and commitment to vaccine uptake [37]. (4) Bilingual translation (traditional Chinese and English) for educational content and MI dialogues was conducted by a Cantonese native speaker with a bilingual translation background and experience. And (5) the coding of educational content into web pages, as well as the coding of educational content-adapted Q&A and MI dialogues into an AI-driven chatbot, was performed by professionals in computer science. This motivational AI-driven chatbot, powered by natural language processing, was embedded in web-based modules to provide real-time, personalized, and interactive conversations on vaccine-related questions.

### 2.4. Step 3: Expert Evaluation

Expert evaluation of the intervention was conducted in March 2023. This evaluation aimed to gather feedback and insights from experts to assess the usability and validity of the intervention content. A panel of five experts was invited to participate in the evaluation. Experts with a Ph.D. degree and at least 5 years of research experience in infectious diseases and/or vaccine research were considered eligible. The experts were provided with access to the AI-driven motivational digital assistant for experience and review within two weeks. A structured questionnaire was developed for expert evaluation covering the following sections [38]: (1) intervention content; (2) platform usability; (3) overall assessment; (4) open questions for suggestions; and (5) socio-demographics, including age, gender, education level, and research experience. For Section 1, Section 2 and Section 3 of this structured questionnaire, a four-point Likert scale (from 1, very inappropriate, to 4, very appropriate) was used to rate scores, and comments were required if the item rating was below 3. The content validity index (CVI) was determined by the average rate of scoring 3 or 4 points to indicate the validity of the intervention content [39]. Expert comments and suggestions were summarized for intervention refinement.

### 2.5. Step 4: Pilot Test

A pilot test was performed in April 2023 to evaluate the feasibility, acceptability, and preliminary effectiveness of the intervention, followed by refinement based on user feedback. We hypothesized that participants would report preliminarily significant improvements in vaccine hesitancy, vaccine-related health literacy, vaccine confidence, vaccine readiness, and vaccination intention post-intervention. The inclusion criteria for pilot test participants were as follows: (1) 18 years or above; (2) vaccine hesitancy (not taking COVID-19 vaccines or receiving involuntary COVID-19 vaccines); (3) have internet access; and (4) able to read Chinese or English. Recruitment involved social media promotion and collaborating with community organizations. After written consent was obtained, eligible participants were provided with access to the AI-driven motivational digital assistant. During the pilot test period, participants received automated weekly emails encouraging them to engage with web-based educational content and interact with the motivational AI-driven chatbot for one module per week. Online questionnaires were performed pre- and post-pilot test.

The questionnaire included the following measurements: (1) socio-demographics; (2) health conditions and COVID-19 infection and vaccination history; (3) vaccine-related health literacy—four items derived from the Chinese version of the European Health Literacy Survey Questionnaire (Cronbach’s α = 0.907) [40]; (4) vaccine confidence—four items measured by the Vaccine Confidence Index (Cronbach’s α = 0.859) [41]; (5) vaccine hesitancy—ten items measured by the Adult Vaccine Hesitancy scale to evaluate vaccine hesitancy (Cronbach’s α = 0.770) [42]; (6) vaccine readiness—one single item, “How ready are you to receive the COVID-19 vaccine?” answered on an 11-point Likert scale from 0 (not at all ready) to 10 (very ready) [43]; (7) vaccination intention—one single item, “Do you plan to take the next dose of COVID-19 vaccine?” with answers of “Yes”, “No”, or “Not Sure”; and (8) two open questions on the limitations of and suggestions for the AI-driven motivational digital assistant.

Data analysis was conducted using the Statistical Package for the Social Sciences 28 [44]. Descriptive statistics were used for summarizing participant characteristics. Pre–post comparisons including the Wilcoxon signed ranks test and chi-squared test were conducted to evaluate the intervention’s effects. Qualitative feedback received from the pilot test as well as expert evaluation were recorded, coded, and discussed to refine the AI-driven motivational digital assistant. This refinement involved addressing usability issues, revising and updating content, and improving the user interface. These refinements aimed to optimize user engagement, satisfaction, and the overall impact of the intervention.

## 3. Results

### 3.1. Step 1: The Development of the Logic Model

The characteristics of the 15 interviewed participants are presented in Table 1. They are from different genders, different age groups, and five nationalities. Four participants were ever infected with COVID-19. Most (n = 10) had already received at least two doses, while only two individuals planned to take another dose. The interview data revealed three themes and nine subthemes related to vaccine hesitancy (see Table 2): personal beliefs, policies and ethics, and the vaccine and vaccination.

**Figure 2:**
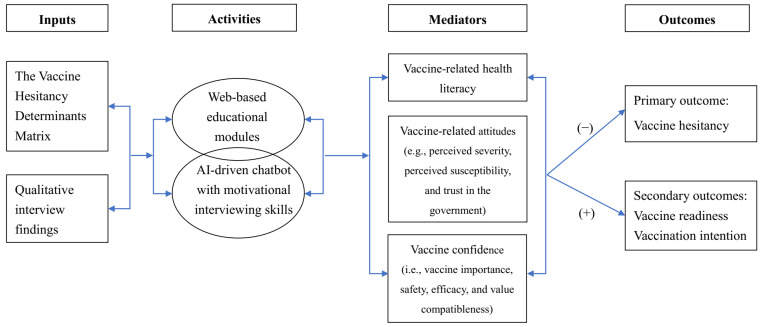
The logical model for the AI-driven motivational digital assistant.

Informed by the Vaccine Hesitancy Determinants Matrix Model and qualitative findings, a logic model was formulated to guide the intervention development. To increase the accessibility of reliable vaccine information/knowledge and the motivation to vaccinate, web-based educational modules and an AI-driven chatbot equipped with MI skills were the core components of the intervention. With mediators of improving vaccine-related health literacy, attitudes (e.g., perceived severity, perceived susceptibility, and trust in the government), and confidence (i.e., vaccine importance, safety, efficacy, and value compatibleness), the intervention was hypothesized to improve the primary (i.e., vaccine hesitancy) and secondary outcomes (i.e., vaccine readiness and vaccination intention) among Hong Kong residents. The logical model for the intervention is presented in Figure 2.

### 3.2. Step 2: The Development of the AI-Driven Motivational Digital Assistant

As guided by the logic model, five module topics were developed collaboratively by the research team. They are as follows: Module 1: Basic Knowledge of COVID-19; Module 2: Basic Knowledge of COVID-19 Vaccine; Module 3: Common Questions about COVID-19 Vaccine; Module 4: Myths about COVID-19 Vaccines; and Module 5: Efforts of the Hong Kong Government. Modules 1 and 2 primarily target enhancing individuals’ vaccine-related health literacy (e.g., finding vaccination information and judging which vaccination is needed) and intervening in vaccine-related attitudes (e.g., perceived susceptibility and severity of complications/long-term effects). Modules 3 and 4 primarily focus on addressing common concerns and misinformation to enhance vaccine confidence, specifically regarding safety and efficacy. Module 5 discusses government policies and ethical considerations aimed at addressing individuals’ distrust in government-provided vaccines and information, as well as improving compatibility with personal values for vaccination. Five to ten multiple-choice questions for each module (Modules 1 to 4) are provided to users to reflect on educational content. Two open-ended questions are provided in Module 5 to collect feedback from participants regarding government efforts and suggestions.

Each module is embedded with a motivational AI-driven chatbot that provides MI dialogues for users to reflect on educational content, perceptions, and willingness regarding vaccination. Module 1 primarily explores individuals’ perceived severity and susceptibility to COVID-19 infection and perceived available health services. Module 2 primarily explores ambivalence towards vaccination and perceived pros and cons as experienced by individuals, and it evokes their intrinsic awareness of the importance of vaccination. Module 3 focuses on identifying information selection bias and personal health beliefs, as well as evoking the importance of vaccination. Module 4 targets misinformation and aims to empower informed vaccine decision making by providing evidence-based information. Module 5 aims to enhance awareness of ongoing efforts and facilitate informed decision making and planning for vaccination with a summary of the five modules. In addition to MI dialogues, Q&As were adapted from educational module contents and integrated into the chatbot to provide real-time responses to vaccine-related questions.

**Figure 3:**
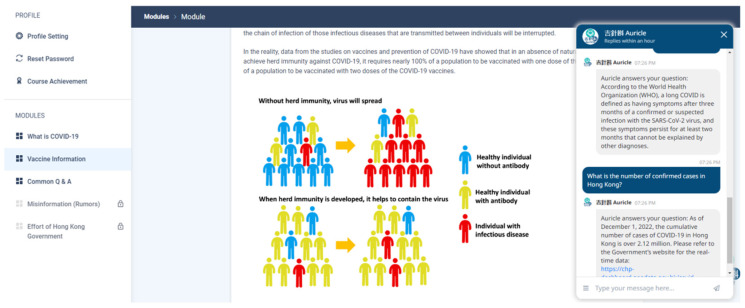
Demonstration of intervention.

After self-learning the educational content of each module, achieving a correct rate of over 80% on multiple-choice questions, and completing MI dialogues with the motivational AI-driven chatbot within a week, a new module was released to users for completion in the following week. Table 3 displays the module contents of the AI-driven motivational digital assistant. Figure 3 demonstrates the interface of the AI-driven motivational digital assistant.

### 3.3. Step 3: Expert Evaluation

The intervention contents were evaluated by five experts (80% female). They were aged 30–49 years old. Research/clinical experience ranged from 5 to 15 years in areas of infectious disease prevention and control, as well as vaccine development and efficacy. A satisfactory averaged CVI of 85.35% was achieved with a range of 78.57% to 95.24%. Experts highlighted the strengths of this digital intervention such as comprehensive vaccine information and engaging visuals. Suggestions were also provided for further refinement in layout, interface clarity, simplifying long-text content, and supporting vaccine efficacy with more informative data and comparisons.

### 3.4. Step 4: Pilot Test

Table 4 shows the characteristics of 12 participants for the pilot test. The sample is predominated by females (66.7%), aged 18–29 years old (91.7%), with college or above educational background (100%), currently unemployed (83.3%), receiving HKD 39,999 or below in monthly household income (91.7%), with an absence of chronic illness (83.3%), and with self-reported good health status (83.3%). Five of them had ever been infected with COVID-19 and eleven had taken three doses of COVID-19 vaccines.

Table 5 presents comparisons between pre- and post-outcome assessments. Significant increases were detected for vaccine-related health literacy (*p* = 0.021) and vaccine confidence (*p* = 0.027). Although significant improvements were not suggested for other outcomes, increased vaccine readiness was observed. Also, fewer users indicated no plan to take the next dose. The users showed moderate satisfaction with this AI-driven motivational digital assistant with a score of 19.75 (SD = 1.14) (range 8–32).

Table 6 presents the correct rate of multiple-choice questions and ratings derived from the motivational AI-driven chatbot for each module. Users achieved high correct rates (89.33% to 98.33%) after learning the educational content of Modules 1 to 4. In general, increasing trends were observed in ratings of vaccine knowledge confidence (from 6.2 to 8.0), vaccine importance (from 6.6 to 7.2), and vaccine readiness (from 6.1 to 7.1). For the feedback collected by open questions, participants expressed high satisfaction with the program. Users found the intervention helpful in addressing their concerns and providing valuable knowledge on COVID-19 vaccines. They appreciated the engaging communication style of the motivational AI-driven chatbot, clear navigation, measurable evaluations, and bilingual modes. Users also provided suggestions for improvement. Some users recommended incorporating additional interactive features or multimedia elements to enhance user engagement. Others suggested strengthening the intelligence of the chatbot to improve interaction and engagement. Based on the findings from the pilot test phase, iterative refinements were made to the intervention by professionals in computer science.

## 4. Discussion

### 4.1. Principal Findings

The AI-driven motivational digital assistant developed in this study was one of the first digital approaches for decreasing vaccine hesitancy in the context of the COVID-19 pandemic. Following systematic processes guided by the Medical Research Council’s framework, this digital assistant was developed with five web-based educational modules that include an embedded motivational AI-driven chatbot. The program’s development was theory-driven and evidence-based with in-depth qualitative interviews, allowing for the identification of factors influencing vaccine hesitancy in the specific context and guiding the generation of tailored content. The expert evaluation demonstrated that the program content was comprehensive and validated. The pilot test revealed that the program was acceptable for usability, and it indicated preliminary effectiveness as well as identified refinement issues that have been addressed accordingly.

The intervention development was theory-driven as underpinned by the Vaccine Hesitancy Determinants Matrix model. The theoretical model provided a comprehensive framework for identifying factors at various levels influencing vaccine hesitancy [4]. Qualitative interviews in this study further contributed to the evidence-based identification of contextual factors, such as cultural factors and policy issues, which can be tailored to address vaccine hesitancy among Hong Kong residents. A logic model that combined insights from the theoretical model and qualitative findings was therefore developed, allowing us to illustrate the mechanism through which the intervention would reduce vaccine hesitancy [27]. Specifically, the intervention targeted influencing factors identified in this study, including vaccine-related health literacy and attitudinal factors, as mediators to reduce COVID-19 vaccine hesitancy and improve vaccine uptake consequently.

Furthermore, expert evaluation provided valuable feedback for content validation, ensuring that the intervention designed to address vaccine hesitancy was effective and user-friendly. The pilot test conducted in this study also supported the preliminary effectiveness of this program and indicated good feasibility and acceptability for its use among the general public. Although a significant improvement in vaccine hesitancy was not detected, the intervention demonstrated preliminarily significant increases in the key predictors (i.e., vaccine-related health literacy and vaccine confidence) of vaccine hesitancy. These positive changes, induced by educational content and AI chatbot-delivered MI dialogues, theoretically contribute to a reduction in vaccine hesitancy [45]. Promisingly, positive trends were observed in vaccine confidence, vaccine importance, and vaccine readiness with chatbot-delivered assessments, indicating an improvement in vaccine acceptance. Users indicated general satisfaction with the useful information and engaging communications offered by this AI-driven motivational digital assistant. Feedback and suggestions were combined to refine the program and sensitively address users’ concerns through a co-designed approach during the iterative development of the toolkit [46].

### 4.2. Study Strengths

The intervention developed in this study was guided by the Vaccine Hesitancy Determinants Matrix model, which addressed one of the limitations of previous studies by providing a comprehensive theoretical underpinning [12,47]. In addition, qualitative interviews conducted in this study provided valuable implications for designing educational content that went beyond addressing the common concerns indicated in previous studies, such as vaccine safety, efficacy, and misinformation [10,18,47]. New components, such as information about coronavirus variants and long-term effects to increase perceived infection severity and government efforts to improve trust level, were identified and integrated into the program to address vaccine hesitancy among people facing similar challenges. The module content developed in our study targets the general public and also highlights a variety of priority populations (e.g., the elderly, pregnant women, and individuals with underlying medical conditions) who are vulnerable to experiencing severe consequences from COVID-19 infection. It expands the potential user base of this program and also caters to the special needs of vulnerable populations, in comparison to earlier studies that focused on specific groups of people [11,47].

In addition to the educational content, one particular strength of our intervention is incorporating MI skills into the AI-driven chatbot. Previous studies have demonstrated the high potential of MI skills to enhance self-efficacy for behavior change, such as addressing vaccine hesitancy [19]. Chatbots are also suggested to facilitate positive attitudes toward COVID-19 vaccines and intention to be vaccinated with advantages in accessibility, information trustworthiness, and interactive experience [22]. Our intervention may provide a more comprehensive and effective solution targeted at vaccine hesitancy by equipping the AI-driven chatbot with MI skills. Through therapeutic dialogues in the chatbot supporting empathy, a sense of personal agency, and evidence-based information, participants are provided with relevant and tailored information to clarify ambivalence and are motivated to make favorable decisions regarding vaccination [37]. As an AI-driven digital assistant, this chatbot can be trained through numerous interactive conversations with users to achieve a more advanced and humanized performance [48].

### 4.3. Implications for Future Work

This study developed and validated an AI-driven motivational digital assistant that incorporated web-based educational content and an embedded motivational AI-driven chatbot, providing an easily accessible, personalized, and supportive e-platform for the general public to address vaccine hesitancy. The study’s findings lay a good foundation for the subsequent fully powered randomized controlled trial. Future studies are recommended to compare the cost-effectiveness of an AI-driven motivational digital assistant versus human-delivered MI interventions in reducing vaccine hesitancy. Although the intervention development was contextualized within a specific geographical population and the COVID-19 pandemic, the systematic intervention development processes demonstrated a valid and comprehensive approach in developing digital interventions for reducing vaccine hesitancy among adult populations. This approach could be applicable to a variety of existing vaccines and, particularly, to future newly developed vaccines in addressing vaccine-preventable diseases.

### 4.4. Limitations

Several limitations need to be acknowledged in this study. Firstly, only one round of expert evaluation was employed, although a satisfactory CVI was achieved. Secondly, study participants with a higher proportion of females may limit the representativeness of the study’s findings for balanced gender perspectives. The small sample size and the dominance of young adults in the pilot test may hinder its representativeness and generalizability to other age populations, particularly older adults who may face challenges in using web-based interventions and communicating with chatbots. Although efforts have been devoted to making the program visually appealing and user-friendly for the elderly, its acceptability among older adults remains to be examined. Thirdly, the lack of longitudinal follow-ups on vaccine hesitancy and related factors limits the evaluation of the long-term effects of this program. Lastly, this intervention is developed based on the sample of Hong Kong residents within the context of the COVID-19 pandemic. The generalizability to other populations and pandemics is not currently demonstrated.

## 5. Conclusions

We developed an AI-driven motivational digital assistant based on the Vaccine Hesitancy Determinants Matrix model and in-depth qualitative interviews. The intervention content was validated by expert evaluation and demonstrated with feasibility, acceptability, and preliminary effectiveness to enhance vaccine-related health literacy and vaccine confidence by a pilot test. This study lays a foundation for conducting a randomized controlled trial to further examine the intervention’s effectiveness. It also sheds light on developing interventions to facilitate informed vaccine decision making and to cope with vaccine-preventable diseases.

## Figures and Tables

**Table 1:** Demographic summary of interview participants (n = 15).

Characteristics	n (%)	Characteristics	n (%)
Age		Gender	
18–29 years	5 (33.3)	Male	4 (26.7)
30–49 years	4 (26.7)	Female	11 (73.3)
50–59 years	4 (26.7)	COVID-19 infection	
60 or above	2 (13.3)	Yes	4 (26.7)
Nationality		No	11 (73.3)
Chinese	11 (73.3)	Vaccination dosage	
Others ^1^	4 (26.7)	Zero doses	4 (26.7)
Plan to take a next dose		One dose	1 (6.7)
Yes	2 (16.7)	Two doses	7 (46.7)
No	6 (50)	Three doses	3 (20)
Not sure	4 (33.3)		

^1^ Others: Palestinian, British, French, and Indian.

**Table 2:** Themes and subthemes of the qualitative interview.

Themes	Subthemes	Quotes
Personal beliefs and bias	Perceived credibility of COVID-19 vaccine information and selection bias	“*Opposed vaccination due to a lack of research to prove the efficacy and safety.*” (P5)
	Religion and cultural factors	“*A friend thought he was blessed by God, so he will not die if infected with the virus.*” (P11)
	Perceived low risk of COVID-19 infection	“*There are only a few cases in my community.*” (P5)
Policies and ethical considerations	Perception of diminished human rights	“*People’s freedom of choice was violated.*” (P3)
	Injection method and setting	“*I’m a little bit scared about the needle.*” (P8)
Vaccine and vaccination	Lack of evidence of COVID-19 vaccine research and development	“*Vaccine was developed in such a short time without any observation.*” (P6)
	Bias about COVID-19 vaccine manufacturing companies and places	“*Manufactures in Western countries are better, and I have less confidence in taking vaccines made in the mainland.*” (P4)
	Side effects of the COVID-19 vaccine	“*It is an unknown for long-term side effects*” (P9)
	Injection method and setting	“*I’m a little bit scared about the needle.*” (P8)

**Table 3:** Module contents of AI-driven motivational digital assistant.

Themes	Subthemes	Quotes
Module 1: Basic Knowledge of COVID-19	COVID-19 and variants Symptoms and complications Long-term effects Vulnerable population Mode of transmission Viral infection detection methods	Explore the perceived severity Explore the perceived susceptibility Explore the perceived available health services
Module 2: Basic Knowledge of COVID-19 Vaccine	Vaccine popularization Vaccine development process Available vaccines in Hong Kong Data-driven vaccine efficacy Priority population and vaccination	Explore ambivalence to vaccination Explore the pros and cons of vaccination Evoking the importance of vaccination
Module 3: Common Questions about COVID-19 Vaccine	Q&A pre-vaccination like age limit, priority group, safety, and efficacy Q&A at vaccination like dosage and mixed vaccination Q&A post-vaccination like side effects and tips for medication	Identify information selection bias Explore personal beliefs and their impact on vaccine decisions and health Evoking the importance of vaccination
Module 4: Myths about COVID-19 Vaccines	Common myths/rumors regarding vaccine safety like plausible side effects Common myths/rumors regarding vaccine efficacy	Identify myths and misinformation Providing evidence-based information Evoking informed vaccine decision making
Module 5: Efforts by the Hong Kong Government	Efforts by the Hong Kong government during the COVID-19 pandemic Input of medical resources Financial and other assistance Humanistic care	Aware of the ongoing efforts to protect public health Summarize the five modules and make an informed decision and plan

**Table 4:** Characteristics of pilot test participants (n = 12).

Characteristics	n (%)	Characteristics	n (%)
Gender		Chronic illness	
Male	4 (33.3)	Yes	1 (8.3)
Female	8 (66.7)	No	11 (91.7)
Age		Self-reported health status	
18–29 years	11 (91.7)	Good	10 (83.3)
30–49 years	1 (8.3)	Fair	1 (8.3)
Educational level		Bad	1 (8.3)
College or above	12 (100)	Infection history	
Monthly household income		Yes	5 (41.7)
HKD 20,000 or below	7 (58.4)	Probably	1 (8.3)
HKD 20,000–39,999	4 (33.3)	No	5 (41.7)
HKD 80,000 or above	1 (8.3)	Prefer not to say	1 (8.3)
Employment		Vaccination dosage	
Employed	2 (16.7)	Two doses	1 (8.3)
Unemployed	10 (83.3)	Three doses	11 (91.7)

**Table 5:** Comparisons between pre- and post-tests [n (%)/M (P25, P75)].

Characteristics	Pre (n = 12)	Post (n = 12)	Z/χ^2^	*p*
Vaccine-related health literacy ^1^	9.5 (8, 12)	12 (9.5, 12)	−2.316	0.021
Vaccine confidence ^2^	11 (9.25, 12.75)	13 (12, 14.75)	−2.209	0.027
Vaccine hesitancy ^3^	29 (24.75, 31)	30 (29, 31.75)	−1.118	0.264
Vaccine readiness ^4^	2 (0.25, 4)	2 (1, 6)	−1.807	0.071
Plan to take a next dose, n (%)			0.202	0.653
Yes	0	0		
No	9 (75.0)	8 (66.7)		
Not sure	3 (25.0)	4 (33.3)		

^1^ Range 4–16. ^2^ Range 4–20. ^3^ Range 10–50. ^4^ Range 0–10.

**Table 6:** Multiple-choice questions and ratings in AI-driven chatbot [mean score].

Characteristics	Module 1	Module 2	Module3	Module 4	Module 5
Multi-choice questions ^1^	98.33	89.33	98.18	90.91	N/A
Knowledge confidence ^2^	6.2	7.0	7.4	7.6	8.0
Vaccine importance ^2^	6.6	7.5	7.3	7.7	7.2
Vaccine readiness ^2^	6.1	6.4	6.2	6.6	7.1

^1^ Range: correct rate 0–100%. ^2^ Range 0–10. N/A for open question.

## Data Availability

The data that support the findings of this study are available from the corresponding author upon reasonable request.
